# Pesticide exposure, risk factors and health problems among cutflower farmers: a cross sectional study

**DOI:** 10.1186/1745-6673-2-9

**Published:** 2007-09-18

**Authors:** Jinky Leilanie Del Prado-Lu

**Affiliations:** 1 National Institutes of Health, University of the Philippines, Manila, Philippines

## Abstract

This was a cross-sectional study which aimed to determine associations between hematologic indices such as red blood cell cholinesterase (RBC) and mean corpuscular volume (MCV), with illnesses related to pesticide exposure among cutflower farmers in La Trinidad, Benguet. One hundred two (102) randomly selected cutflower farmers underwent comprehensive, personal physical health and laboratory examinations and answered a questionnaire on work practices and illness. Majority were males (52%) and most belonged to the 20–35 age group (45%). Majority of exposed farmers were symptomatic, with most common complaints being headache (48%), easy fatigability (46.1%) and cough (40.2%). Analysis showed that RBC cholinesterase levels were positively associated with age (p = 0.02), and selling pesticide containers (p = 0.008). number of years of using pesticides (p = 0.022), use of contaminated cloth (p = 0.033), incorrect mixing of pesticides (p = 0.041), sex (p = 0.002) and illness due to pesticides (p = 0.005) were correlated with abnormal MCV. Significant associations were also found for hemoglobin, hematocrit, RBC, white blood cell (WBC) and platelet count. Predictors of RBC cholinesterase were years of pesticide use (p = 0.037) and abnormalities on health (p = 0.029). The findings of the study can be used for information dissemination and pesticide reduction programs for the cutflower farmers.

## Background

Agriculture is a basic source of income and subsistence among many Filipinos. Despite the rise of industrialization, agriculture remains a highly significant contributor to the country's Gross Domestic Product. One of the leading sectors in agriculture in terms of income and growth is the local cutflower industry. Unknown to many, the Philippine flower industry provides a significant portion of earnings derived from agriculture. It has become a lucrative business and much of the country's supply comes from the flower plantations in La Trinidad, Benguet. This municipality grows cutflowers like roses, mum, chrysanthemums, angel's breath and anthorium, accounting for a billion dollar industry. Due to the steep competition and large demand, many farmers resort to the extensive use of pesticides to increase yield.

Pesticide use has been documented to lead to adverse health effects. Pesticide related health problems usually manifest as a series of symptoms depending on severity of exposure. For instance, mild organophosphate poisoning manifests in the form of malaise, vomiting, nausea, diarrhea, loose stools, sweating, abdominal pain and salivation. Moderate poisoning includes dyspnea, decreased muscular strength, bronchospasm, miosis, muscle fasciculation, tremor, motor incoordination, bradycardia, and hypotension/hypertension. Severe manifestation could result in coma, respiratory paralysis, extreme hypersecretion, cyanosis, sustained hypotension, extreme muscle weakness, muscular paralysis and convulsion (Iowa State University, 1995)[1]. Other illnesses associated with pesticide exposure are dermatitis, asthma exacerbation (Sanborn, Cole, Abelsohn, Weir, 2002)[2], sensory peripheral nerve defects, chronic neurobehavioral and motor dysfunction (Miranda, McConnell, Delgado, Cuadra, 2002;, Miranda, McConnell, Delgado, Cuadra, 2004) [3,4], deficits in verbal abstraction, attention, and memory (Farahat, Abdelrasoul, Amr, Shebl, 2003)[5], and anxiety and depression (Jamal, Hansen, Pilkington, Buchanan, 2002)[6]. These effects have been suggested to impair farmers' ability to comply with established safety procedures (Beseler and Stallones, 2003)[7].

This study aims to determine association between hematologic indices such as RBC cholinesterase and mean corpuscular volume (MCV), and illness among cutflower farmers. This is the first ever data for cutflower farmers in the Philippines. Biological marker such as monitoring of serum cholinesterase and cholinesterase enzymes in red blood cells (RBC) can assess actual exposure to pesticides particularly organophosphates (Tayser, 2005) [8]. Organophosphates inhibit the action cholinesterase thus increasing the cholinergic effects of the neurotransmitter, acetylcholine in the body and depolarization of neural transmission (Weiss, Amler S, Amler R, 2004) [9]. Below 50% from the baseline data of serum cholinesterase indicates a significant acute organophosphate toxicity.

Data from this study can be helpful in formulating medical surveillance for farmers and to improve working conditions in the cutflower industry by formulating an integrated program on safe and healthy work practices.

## Methodology

An initial situational analysis was conducted to investigate the nature and method of pesticide use and application which included the brand of pesticide, the active ingredients, and the concentration of the mixture and the individual component. A cluster multistage sampling ter4chniqyue was done. A total of 102 subjects were chosen, with level of significance at *P *= .05.

The study was cross sectional since all the barangays in La Trinidad, and the entire province are engaged in either cutflower or vegetable farming commercially. A comparable control group with pesticide exposure as the variable would be difficult to identify within the area. The target area is also much higher in altitude than the rest of Luzon Island which makes certain physiologic profile of farmers different. The agricultural crops grown between the lowland and highland would also be different, and thus, would have some disparity in terms if pesticide use. Although cross sectional study is inferior to case control in identifying the risk factors to health effects, the study tried to differentiate between exposed (directly) and unexposed within the same subject population.

Data gathering was done using the following:

1. Questionnaire – Interview with farm workers/farmers was done. Details included personal information, health history, pesticide usage, work practices, work conditions, other risk factors and health data.

2. Exposure assessment monitoring – Blood cholinesterase activity was also determined for each farmer of possible effect of pesticides in the biological system.

3. Individual physical health assessment was done by the medical doctors who were part of the implementation of the project.

4. Laboratory examinations, including blood extraction for RBC cholinesterase levels, complete blood count, and kidney and liver function tests were done by a licensed medical technologist. Organophosphate poisoning can be indicated by a decrease in RBC cholinesterase.

5. Work analysis in each farm to validate work practices related to pesticide preparation and application.

The specific factors studied in relation to health problems included pesticide-related symptoms, the categories of illness refer to symptoms rather than specific illnesses. Acute symptoms include vomiting, eye irritation, headache, nausea and allergic reactions. The more chronic symptoms include imbalance in gait, tearing of the eye, chronic dermatitis, neurologic problems, or even cancer. Five (5) ml of blood was extracted and placed in a heparin tube for blood cholinesterase determination. An informed consent was given to participants.

The biologic and physiologic correlates of pesticide exposure included blood cholinesterase level, and the symptoms and illnesses experience by the respondents. Data were analyzed using SPSS 10.0

## Results and Discussion

### a. Socio-demographic profile

Majority of the respondents were males (52%) while 48% were female. None of the women was pregnant at the tine of the study. Most belonged to the 20–35 age group (45%), with ages ranging from 15 to 68 (mean age is 36.4 ± 13.09). this shows a population in their middle adult years. Majority cultivated roses in their farms (36.4%) while 5% grew mums.

### b. Medical history

Hypertension was the most prevalent illness reported among the respondents (13%) and their families (26.4%), followed by allergy (6.7% and 5.3% for respondents and families respectively) and asthma (3.4% and 10.1% for respondents and families respectively). Of 380 reported pregnancies, 20 (5.26%) were preterm while 6.3% were abortions. Two cases of congenital anomalies were also found (Table 1). It has also been found that infertility is more common in women involved in agriculture and those who live in farms (Fuortes, Clark, Kirchner, Smith, 1997) [10]. The study of Beam in 2004 [11] reported that babies born to women with high levels of pesticides in their blood are lighter than babies who had not been exposed to the chemicals. In China's rural Anhui province (Raloff, 2004) [12], it was indicated that at DDT concentrations present in young women there, the pesticide can affect both menstrual cycles and can cause miscarriages in the first few weeks of pregnancy.

**Table 1:** Medical History of Cutflower Farmers and their Families (N = 102)

**Disease**	**Past History**	**Family History**
	
	**Percentage**	**Percentage**
Hypertension	13.0	26.4
Diabetes mellitus	2.4	5.8
Ischemic heart disease	3.4	5.8
Kidney disease	2.9	5.3
Asthma	3.4	10.1
Allergy	6.7	5.3
Cancer (leukemia, osteosarcoma)	1.0	1.9
Endocrine (goiter)	1.0	1.9
Wife's Obstetric History (N = 380)		
Full term	88.4	
Preterm	5.26	
Abortion	6.3	
Congenital anomalies		

Alcohol drinking was common among the respondents (50.5%) while cigarette smoking was reported by 25.5% of respondents. Betel chewing was reported by 11.5% of the respondents. Majority used gas stoves and microwaves for cooking, while deep well was the predominant water source (26%). The diet of the farmers consisted mainly of vegetables (55.8%) followed by seafood (except fish) and seaweed.

### c. Pesticide use

Among the pesticides used, the most toxic and hazardous is Dithane, which is a category IV pesticide containing Mancozeb as its active ingredient (Table 2). Dithane has been used by 35.1% of farmers for approximately 11 years with the mean usage of 1000 ml per pesticide usage. Tamaron is a category II pesticide containing the organophosphate Methamidophos and Diethylene glycol, while Lannate is a category Ib pesticide whose active ingredient is Methomyl. A study done locally by Baurdoux, Snelder, De Snoo, in 2004 [13] also found prevalent use of and easy access to pesticides classified by the WHO as highly or moderately hazardous and some pesticides tagged for restricted use by Environmental Protection Agency among farmers in the Cagayan Valley.

**Table 2:** Number of Cutflower Farmers using Certain Pesticides and Quantity Used (in Volume); N = 102*

**Brand Name of Pesticide**	**Generic Name of Pesticide**	**WHO Category**	Number**	**Percentage**	**Mean number of years**	**Mean amount used (mL)**
Tamaron	Metaldehyde	II	42	20.2	17.11	106.92
Lannate	Methiocarb	Ib	72	34.6	11.67	347.35
Dithane	Mancozeb	IV	73	35.1	11.16	1,185.6
Selecron	Profenofos	III	64	30.8	9.13	136.40
Agrimix	Avermectin	II	47	22.6	7.01	251.67
Matador	Methamidophos	Ib	25	12.0	7.96	66.92
Basudin	Diazinon	II	15	7.2	7.93	557.67
Karate	Lambdacyhalothrin	IV	12	5.8	6.92	290.42

* Respondents had multiple answers

** Number refers here for the number of cut flower farmers who used this kind of pesticide.

### d. Pesticide exposure

Certain behaviors and practices were identified to predisposed to pesticide exposure and illness. Twenty percent (20%) of the farmers used pesticides for more than 20 years and almost 15% have used it for 11–20 years. This is very significant, and indicates chronic exposure among these farmers. The farmers were exposed to 30 minutes to 4 hours per day every application, with an average of 3 hours. They are exposed about 1 to 4 days a week or an average of one and a half days in the application of pesticide.

The activities performed by the farmers while working with pesticides were loading, applying, and mixing (76.4%, 77.4% and 76.4% respectively). During these activities, they are exposed for more than 12 times a year, which is quite considerable. Incorrect work practices were also noted among farmers such as re-entering recently sprayed area (79.3%), wiping sweat off the face (66.8%), spraying against the wind (23.1), spills at the back (45.2%) and while spraying (51.9%), loading (29.8%) and mixing (35.1).

Despite the high risk and frequency of exposure, farmers did not wear proper personal protection while working with pesticides. Boots were the only protective equipment worn by majority of the farmers, and practically no one used aprons or gauntlet gloves. Cloth face masks which do not offer adequate coverage for some chemicals were used by a number of respondents (41%). Improvise forms of PPE were also used such as handkerchiefs, long sleeves and plastic pants.

Re-entering a recently sprayed area has been the cause of a poisoning outbreak in Poland in 2002 after applicators re-entered a contaminated area before the required safety period has lapsed. In the same country, 22 poisoning cases were seen as a result of spraying without adequate protective gear (Przybylska, 2004) [14]. This shows the seriousness of the situation faced by the farmers. When it comes to disposal of pesticide containers, majority (32.4%) said that they stored used containers in their backyard. This is a dangerous practice since household members may mistake it for another container and reuse it. Other previously identified risk behaviors for exposure included frequent pesticide use, washing pesticides equipment in water sources used by humans, inadequate disposal of empty pesticide containers, and eating and drinking during pesticide application (Hurtig, San Sebastian, Soto, Shingre, 2003) [15].

### e. Pesticides and health

#### e1. Clinical manifestations

A number of respondents (23.5%) reported being ill due to pesticide use during the last 12 months, with 2.9% having constant illness, 3.9% having frequent illness and 16.7% exhibiting occasional symptoms. Among the ill, only one reported always seeking medical advice in times of illness, while 7% said that they only consulted occasionally. Onset of illness was reported to be after pesticide use.

Pesticides have been associated with a number of diseases, and even death. This was seen by Fleming, Gomez-Martin, Zheng, Ma, Lee, et al. in 2003 [16], who studied mortality linked 1986–1994 National Health Interview Survey data. They found that farmers and pesticide applicators were at greater risk of accidental mortality compared to all other workers. Furthermore, both male and female workers had a higher risk of cancers of the nervous, lymphatic and hematopoietic systems. Among infants, Young, Eskenazi, Gladstone, Bradman, Pedersen, Johnson, Barr, Furlong, Holland, (2005) [17] documented a significant association between *in utero *organophosphate exposure and abnormal reflexes, which may be associated with subsequent impairment of neuropsychological functioning. Lander and Ronne (1995) [18] also found significant odds ratio for leukemia among farmers. These point out the role of pesticides in carcinogenesis and disruption of hematopoiesis. Genotoxicity has also been linked to pesticides (Undeger & Basaran, 2005; Varona, Cardenas, Crane, Rocha, Cuervo, Vargas, 2003) [19,20].

General symptoms (weakness, fever, lethargy) were the predominant abnormal manifestations among those examined (63.8%). HEENT symptoms (blurring of vision, deafness, headache) were also predominant among the farmers. Involvement of the skin was also noted, with 21% of farmers having integumentary abnormalities. Specifically, headache was the most frequently reported symptom (48%) closely followed by easy fatigability (46.1%) and cough (40.2%). Blurring of vision and palpitations were also common (36.3% and 33.3% respectively). Similar symptoms were found by Strong, Thompson, Coronado, Griffith, Vigoren, Islas, in 2004 [21] among farmers exposed to organophosphates.

On physical examination, 90 or 88.2% of those examined were found to have abnormal peak expiratory flow rate (PEFR). Eighty two percent had abnormal temperature, followed by abnormal health findings (e.g. cardiorespiratory distress). Forty one percent were also found to have elevated blood pressures (Table 3). Such a constellation of symptoms are consistent with previous findings of increased likelihood of chronic disability, health conditions, and poor health among pesticide applicators (NPCIS, 2004) [22].

**Table 3:** Frequency Distribution of Abnormal Physical Examination among Cutflower Farmers (N = 102)

**Abnormal Physical Examination**	**Normal Values/Normal Indices**	**Number**	**Percentage**
Peak expiratory flow rate (PEFR)	Obstructive or restricted lungs using spirometry; difficulty in respiration	90	88.2
Temperature	Not within 36.5–37.5 degrees C	84	82.4
Throat	Abnormal growths or lumps	59	57.8
Extremities	Abnormal growth or lumps	50	49.0
Blood pressure	Not within 120/80 mmHg for females; 90/60 for females	42	41.2
Eyes	Abnormal growths or lumps, redness and tearing of the eye	37	36.3
Head	Abnormal growth or lumps	27	26.5
Ears	Abnormal growth or lumps	17	16.7
Neck	Abnormal growth or lumps	17	16.7
Heart rate	600–100 beats per minute	16	15.7
Lungs	Abnormal growth or lumps	4	3.9
Heart	Abnormal murmurs and sounds with stethoscope	2	2.0
Abdomen	Abnormal growth or lumps	2	2.0
Nose	Abnormal growth or lumps, clogging, inflammation	1	1.0

#### e2. Laboratory examinations

Cholinesterase actually corresponds to two enzymes – acetylcholinesterase and butyrylcholinesterase (also called plasma cholinesterase) (Hernandez, Gomez, Pena, Gil, Rodrigo, Villanueva, Pla, 2004) [23]. The activity of cholinesterase enzymes in the blood can be utilized as a biomarker for the effect of organophosphates. An exposed person will show abnormally low levels of activity of cholinesterase enzymes measured in the serum or in red blood cells (as RBC cholinesterase). The latter is more closely correlated with cholinesterase activity in the nervous system (Tinoco-Ojanguren & Halperin, 1998) [24].

It should be noted, however, that RBC cholinesterase is more difficult to measure and is depressed more slowly than plasma cholinesterase. Certain pesticides also exhibit preferential inhibition of either enzyme. Hence, levels of both enzymes should be determined to accurately determine pesticide exposure (Boiko, Keifer, Furman, Weyrauch, Hanks, 2005) [25].

In Sitio Sadag, 51% had cholinesterase levels below the mean value of 0.75–1.0 Δ ph/hour, and 25.5% exhibited more than 10% depression in the level of RBC cholinesterase. Tinoco-Ojanguren and Halperin in 1998 [24] also found similar lowering of cholinesterase values among agricultural peasants. Ceratin hematological parameters wee also abnormal, namely hemoglobin, hematocrit, and eosinophil count. These laboratory findings are similar to those found by Svoboda [26] in 2001. The liver (ASL and LAT) and kidney function test (creatinine) were all normal for the respondents (Table 4).

**Table 4:** Frequency distribution of Abnormal Laboratory Examination Results of Cutflower Farmers

		**Abnormal Results**	
**Laboratory Examination**	**Normal Values**	**Number**	**Percentage**

Hemoglobin	120–180 g/L	16	15.7
White blood cell count	4–11 × 109 g/L	35	34.3
Hematocrit	0.370–0.540	22	21.6
Platelet count	150–450 × 109 g/L	4	3.9
Aspartate Transaminate (AST)	15–37 units/L	13	12.7
Analine Transaminate (ALT)	30–65 units/L	25	24.5
Creatine	53–155 umols/L	21	20.6
RBC Cholinesterase	Δ ph/hour 0.75–1.0 ph/hour	52	51.0
% Depression of RBC Cholinesterase	0.75–1.0 Δ ph/hour	26	25.5

### f. Chi square test of independence

After performing chi-square analysis to test for independence, significant association was found between selling pesticide containers and abnormal RBC cholinesterase levels (P = 0.001), and mixing of pesticides with abnormal mean corpuscular volume (MCV) (Table 5).

**Table 5:** Chi-Square Association between Incorrect Work Practices and Abnormal Laboratory Findings (RBC Cholinesterase and Mean Copuscular Volume) (N = 102)

	**Abnormal RBC Cholinesterase**	**Abnormal Mean Copuscular Volume**
Reuses container to store other things	3.504 (0.061)	35.306 (0.083)
Sells the container	10.829 (0.001)	
Mixing of pesticides		40.549 (0.05)

Cholinesterase measurements also have limitations, since the rate of enzyme inhibition and subsequent recovery may differ with exposure to varying organophosphates. Cholinesterase levels are also affected by inter- and intra-individual variability (Tinoco-Ojanguren and Halperin, 1998) [24]. Therefore, pre-exposure baseline levels should be established for each individual so that meaningful changes in cholinesterase levels may be detected (Hernandez, Gomez, Pena, Gil, Rodrigo, Villanueva, Pla, 2004) [23].

In addition, certain conditions other than pesticide exposure can lower plasma and RBC cholinesterase levels, confounding interpretation of test results. The former can be decreased by liver disease, malnutrition, alcoholism, nephritic syndrome, early pregnancy, contraceptive pills, and metoclopramide. Meanwhile, RBC cholinesterase levels are lowered by hemolytic and pernicious anemia, recovery from hemorrhage, and reticulocytosis. Other factors that may result in false cholinesterase levels are collection, shipping and laboratory errors, and poor record keeping and organization (Boiko, Keifer, Furman, Weyrauch, Hanks, 2005) [25].

Many other hematologic changes secondary to acute and chronic pesticide exposure have been documented in both humans and animals, although there are some conflicting results (Meaklim, Yang, Drummer, Killalea, Staikos, Horomidis, Rutherford, Ioannides-Demos, Lim, McLean, McNeil, 2003; Saly, Kacmar, Neuschl, Jantosoovic, 1995) [27,28]. Pesticides have been shown to have hematotoxic properties and may cause aplastic anemia, agranulocytosis, neutropenia, and thrombopenia (Parent-Massin & Thouvenot, 1993) [29]. In rats, Fujitani, Tada, Yoneyama, (2004) [30] found that sub-chronic exposure to chlopropham induced dose-dependent, although reversible methemoglobinemia, anemia, splenomegaly and pathological lesions indicating hemolytic anemia. Irreversible changes were increased hemosiderin deposition and splenic capsular fibrosis. Far more serious and long-term consequences have been seen in humans by Khristeva and Mirchev in 1993 [31]. They found that both acute and chronic exposure to toxic doses of pesticides as well as drugs and heavy metals may induce hematologic congenital abnormalities, particularly G6PD deficiency and thalassemia.

### g. Linear regression analysis

There is a significant positive relationship using linear regression between age and abnormal RBC cholinesterase levels (p = 0.020). An even more significant association was found between abnormal RBC cholinesterase levels and selling pesticide containers (p = 0.008). This is probably because farmers often clean the containers before selling them, thereby exposing themselves to residues present in the containers.

Meanwhile, five variables were significantly correlated with abnormal mean corpuscular volume (MCV). There were the number of years of using pesticides (p = 0.022), improper mixing of pesticides (p = 0.041) and sex (male farmers tended to have a higher MCV at p-0.002), use of contaminated cloth (p = 0.033) and illness due to pesticides (p = 0.005). See Table 6.

**Table 6:** Predictors of Abnormal Mean Copuscular Volume (MCV) of the Cutflower Farmers (N = 102)

**Risk Factors**	**Beta Coefficient**	**Significance**
Years using pesticides	0.244	0.001
Hours of exposure	0.434	0.025
Sex	4.409	0.040
Abnormal blood pressure	2.139	0.221
Abnormal RBC count	5.328	0.039

For abnormal hemoglobin levels (Hgb), significant correlations were found for number of years using pesticides (p = 0.017), not consulting a doctor when sick (p = 0,025), abnormal respiratory rate and sex, indicating that male farmers have higher abnormal Hgb level, and abnormal blood pressure (p = 0.008). Changes in hemoglobin levels as well as electrocardiograms have been previously associated with early hexachlorocyclohexane exposure (Srivastava, Gupta, Bihari, Mathur, Pangtey, Bharti, 1995) [32]. See Table 6. A similar association between RBC count and pesticide use was also reported with hexachlorocyclohexane by Shouche and Rathore in 1997 [33].

Table 7 shows the predictors of abnormal RBC cholinesterase levels and abnormal MCV as dependent variables. The number of years of pesticide use was found to be a highly significant predictor of MCV (p = 0.001). Other predictors are number of hours of pesticide exposure (p = 0.025), sex (p = 0.040) and RBC count (= 0.039). Women usually have lower MCVs than men because they regularly shed blood due to menstruation. Meanwhile, MCV is an index of RBC count, therefore a lower RBC count would result in lower MCVs. Since we have already accounted for possible normal physiological explanations for these results, it seems that pesticide exposure, in particular the length of exposure, is a highly significant predictor of MCV levels. Similar studies, like that of Casale, Scott, Anderson, JR., Vitzthum, Gold, [34], have found that pesticide use is a significant predictor of RBC count and hematocrit and that extensive use of pesticides significantly reduces serum complement activity.

**Table 7:** Predictors of Abnormal Red Blood Cell (RBC) Cholinesterase Levels of the Cutflower Farmers

**Risk Factors**	**Beta Coefficient**	**Significance**
Years using pesticides	2.146	0.037
Abnormal respiratory rate	1.228	0.079
Abnormal health symptoms	6.22	0.029

As for RBC cholinesterase (Table 7), the significant predictors are, again, years of pesticide use (p = 0.037) and health symptoms reported in the survey (p = 0.029). the number of years using pesticides gives an index of the length and possible extent of exposure, which has been shown to lower RBC cholinesterase levels. Health symptoms included in the survey were non-specific such as drowsiness, and may be attributed to a number of conditions. This makes the detection of pesticide exposure/poisoning very difficult since no distinctive or specific symptom is predictive of the event. Further elucidation of clinical manifestations that may be used as predictors of pesticide exposure must be conducted for early and easy detection of possible poisoning. Moreover, differentiation must be made between acute and chronic exposures.

## Conclusion

The study has demonstrated the detrimental effect of pesticide exposure on RBC cholinesterase levels and the association of various hematologic indices with risk factors and measures of pesticide exposure. Abnormal RBC cholinesterase levels were positively associated with age (p = 0.020), and selling pesticide containers (p = 0.008), number of years of using pesticides (p = 0.022), use of contaminated cloth (p = 0.033), and illness due to pesticides (p = 0.005), improper mixing of pesticides (p = 0.041), and sex (p = 0.002). Significant associations were also found for hemoglobin, hematocrit, RBC, WBC and platelet count. Furthermore, number of years of pesticide use (p = 0.000), hours of pesticide exposure (p = 0.025), sex (p = 0.040), and lowered RBC count (p = 0.039) were found to be predictors of abnormal MCV. Predictors for RBC cholinesterase, are years of pesticide use (p = 0.037) and abnormalities on general survey (p = 0.029).

These findings are further proof of the hematoxic effects of pesticide exposure. The risk factors and work behaviors identified in this study could be utilized as a target for modification and improvement of safety practices among cutflower farmers who significantly contribute to the country's growth. A more in depth study is needed to differentiate between acute and chronic effects. It will also be worthwhile to look into specific hematopoietic effects of pesticide use since these have implications for cancer development and possible prevention.
